# Prediction of vertical distribution of SPAD values within maize canopy based on unmanned aerial vehicles multispectral imagery

**DOI:** 10.3389/fpls.2023.1253536

**Published:** 2023-12-18

**Authors:** Bo Chen, Guanmin Huang, Xianju Lu, Shenghao Gu, Weiliang Wen, Guangtao Wang, Wushuai Chang, Xinyu Guo, Chunjiang Zhao

**Affiliations:** ^1^ Information Technology Research Center, Beijing Academy of Agriculture Forestry Sciences, Beijing, China; ^2^ Beijing Key Laboratory of Digital Plant, China National Engineering Research Center for Information Technology in Agriculture, Beijing, China; ^3^ College of Resources and Environment, Jilin Agricultural University, Changchun, China; ^4^ Nongxin Science & Technology (Beijing) Co., Ltd, Beijing, China

**Keywords:** canopy chlorophyll, SPAD values, maize, UAV multispectral, vertical distribution

## Abstract

Real-time monitoring of canopy chlorophyll content is significant in understanding crop growth status and guiding precision agricultural management. Remote sensing methods have demonstrated great potential in this regard. However, the spatiotemporal heterogeneity of chlorophyll content within crop canopies poses challenges to the accuracy and stability of remote sensing estimation models. Hence, this study aimed to develop a novel method for estimating canopy chlorophyll content (represented by SPAD values) in maize (*Zea mays* L.) canopies. Firstly, we investigated the spatiotemporal distribution patterns of maize canopy SPAD values under varying nitrogen application rates and different growth stages. The results revealed a non-uniform, “bell-shaped” curve distribution of maize canopy SPAD values in the vertical direction. Nitrogen application significantly influenced the distribution structure of SPAD values within the canopy. Secondly, we achieved satisfactory results by fitting the Lorentz peak distribution function to the SPAD values of different leaf positions in maize. The fitting performance, evaluated using R^2^ and RMSE, ranged from 0.69 to 0.98 and 0.45 to 3.59, respectively, for the year 2021, and from 0.69 to 0.77 and 2.38 to 6.51, respectively, for the year 2022.Finally, based on the correlation between canopy SPAD values and vegetation indices (VIs) at different growth stages, we identified the sensitive leaf positions for the selected CCCI (Canopy Chlorophyll Index) in each growth stage. The 6th (r = 0.662), 4th (r = 0.816), 12th (r = 0.722), and 12th (r = 0.874) leaf positions exhibited the highest correlations. Compared to the estimation model using canopy wide SPAD values, the model based on sensitive leaf positions showed improved accuracy, with increases of 34%, 3%, 20%, and 3% for each growth stage, respectively. In conclusion, the findings of this study contribute to the enhancement of chlorophyll content estimation models in crop canopies and provide valuable insights for the integration of crop growth models with remote sensing methods.

## 1 Introduction

Chlorophyll is a vital photosynthetic pigment that plays a crucial role in the transfer and conversion of light energy. Its primary function is to absorb light energy and convert it into chemical energy, which enables the photolysis of water and the production of reduced coenzyme NADPH, crucial for the smooth progress of photosynthesis (Croft et al., 2017; Li et al., 2020; Sun et al., 2023). Leaf Chlorophyll Content (LCC) is a critical factor in determining photosynthetic capacity and is commonly used by agronomists to guide nitrogen fertilizer application, as chlorophyll is one of the primary storage units for nitrogen and its content is highly correlated with nitrogen levels (Lemaire et al., 2008; Schlemmer et al., 2013; Zhou et al., 2023). Furthermore, nitrogen deficiency in crops such as maize is often indicated by a decrease in leaf green area and age (Ciampitti and Vyn, 2011; Li Y. et al., 2022). The conventional approach to measuring chlorophyll content involves initially conducting destructive sampling of crops, followed by sending the processed samples to the laboratory for testing. While this method offers high accuracy, it suffers from drawbacks such as time-consuming and labor-intensive processes, as well as data latency. However, a chlorophyll meter developed by Soil Plant Analysis (referred to as “SPAD” hereafter), manufactured by Konica Minolta in Tokyo, Japan, has been utilized to obtain the relative chlorophyll content of leaves (Samborski et al., 2009; Yuan et al., 2016). Compared to the traditional approach, the implementation of the SPAD instrument offers numerous advantages, including enhanced efficiency, non-destructiveness, and freedom from time and environmental constraints. As a result, this study opted to employ the SPAD values as a representative measure of LCC.

The chlorophyll content of leaves demonstrates a conspicuous non-uniform distribution pattern in the vertical direction, which is influenced by the canopy structure characteristics and cultivation mode of maize varieties (Li et al., 2013; Li L. et al., 2022). Therefore, accurately assessing the spatial distribution and temporal changes of chlorophyll content in maize holds significant importance for modern agricultural production. The vertical disparity in plant canopy chlorophyll content (CCC) is commonly represented by a “bell-shaped” distribution (Ciganda et al., 2008; Winterhalter et al., 2012), indicating that the LCC varies among different leaf positions. Additionally, the transfer of nitrogen between leaf layers during different growth stages and fertilization treatments further amplifies the spatiotemporal variability of LCC within the canopy. While current models can predict crop CCC to some extent (Huang et al., 2022; Kushwaha et al., 2022), they often overlook the heterogeneity of LCC distribution in the vertical space, resulting in significant errors during practical application (Winterhalter et al., 2012). Hence, the incorporation of vertical distribution characteristics of LCC into the maize chlorophyll content model is pivotal. This integration facilitates the quantitative depiction of LCC within the canopy and elucidates the temporal and spatial distribution characteristics of chlorophyll content. Consequently, it enhances the timeliness and precision of field production management while safeguarding maize from low nitrogen stress or excessive resource input.

Currently, several studies have represented the vertical non-uniform distribution curve of maize canopy LCC using different functions, such as quadratic functions (Evers et al., 2005) and curve functions based on thermal time (Li L. et al., 2022). However, the mentioned functions fail to incorporate the structural characteristics of the maize canopy, as well as the impact of environmental factors like variety, fertilization, and growth stage. This limitation results in a restricted mechanistic understanding and reduced accuracy of the model. Moreover, crop growth and development lead to variable vertical nutrient distribution, influenced by conditions of nitrogen fertilizer and stages of growth (Yan-Li et al., 2018). Thus, it is vital to take these factors into account during the construction of the LCC model. In previous research, the Lorentz peak distribution function: *y = y_m_/[1 + ((x – x_0_)/b) ²]* Equation 1, a nonlinear regression curve, was successfully utilized to simulate winter barley leaf growth and the relationship between the main stem and tiller leaf length in wheat (Evers et al., 2005), demonstrating favorable performance. The three parameters, *y_m_*, *x_0_*, and *b*, in this function represent the peak value of the curve, the independent variable corresponding to the peak value, and the slope coefficient of the curve, respectively, resembling the vertical non-uniform distribution characteristics of maize canopy LCC. Moreover, the maximum LCC (*y_m_*), the corresponding leaf position (*x_0_*) of maize, and the variation range of LCC across different leaf layers (*b*) have been observed to be influenced by phenological stages and fertilization treatments. However, currently, there is a paucity of research focusing on the fitting of the Lorentz peak distribution function to characterize LCC within the maize canopy.

In recent decades, unmanned aerial vehicle (UAV) spectral remote sensing has emerged as a highly valuable tool for spatial diagnosis and prediction of crop chlorophyll, offering advantages such as high-throughput capabilities, low-cost implementation, and non-destructive assessment (Lang et al., 2019; Wang et al., 2022). With the continuous improvement of technical means, as well as advancements in image temporal and spatial resolution, UAV remote sensing has garnered increasing attention in modern agriculture (Brocks and Bareth, 2018). Numerous studies have employed vegetation indices (VIs) for chlorophyll content monitoring and retrieval. Many studies have utilized vegetation indices (VIs) for monitoring and estimating chlorophyll content, including the widely employed modified red-edge ratio (mRER) and canopy chlorophyll content index (CCCI), which have been demonstrated to exhibit a strong correlation with leaf chlorophyll content (LCC). For instance, (Yang et al., 2022) utilized the mRER index and achieved an optimal model for estimating LCC in maize(R^2^ = 0.87). This model accurately predicts LCC during the middle growth stage. In another study, conducted by (Cammarano et al., 2011) over a three-year experimental period in Australia, the canopy green CCCI successfully predicted the canopy nitrogen (N) content (g m^-2^) of rainfed wheat pre- and post-jointing stage (R^2^ = 0.97, RMSE=0.65g m^-2^). These VIs, calculated based on the spectral reflectance in the visible and near-infrared bands, exhibit a robust association with LCC and can be easily derived from multispectral image data. Currently, the transferability and stability of UAV remote sensing models are constrained by the limited consideration of crop physiological structure information and the failure to integrate the temporal and spatial distribution characteristics of canopy LCC into remote sensing models for chlorophyll prediction.

Understanding the spatiotemporal distribution of canopy LCC is crucial, providing essential insights for precise crop nutrient diagnosis and management strategies at key stages of the growing season. Additionally, this knowledge offers theoretical foundations for chlorophyll estimation through remote sensing imagery. Integration of UAV remote sensing with the canopy chlorophyll distribution function model holds promise for enhancing the accuracy of maize canopy LCC estimation. Therefore, this paper aims to: 1) investigate the spatial and temporal distribution characteristics of canopy LCC in maize during critical growth stages and under different nitrogen fertilizer treatments; 2) employ the Lorentz peak distribution function to quantitatively simulate canopy LCC in maize across various growth stages and nitrogen fertilizer treatments, while elucidating the spatiotemporal variations of function parameters; 3) assess the general applicability of the Lorentz peak distribution function in fitting canopy LCC across different years and evaluate the potential of combining UAV multispectral data with the canopy LCC distribution function.

## 2 Materials and methods

### 2.1 Study site and experimental design

**Figure 1:**
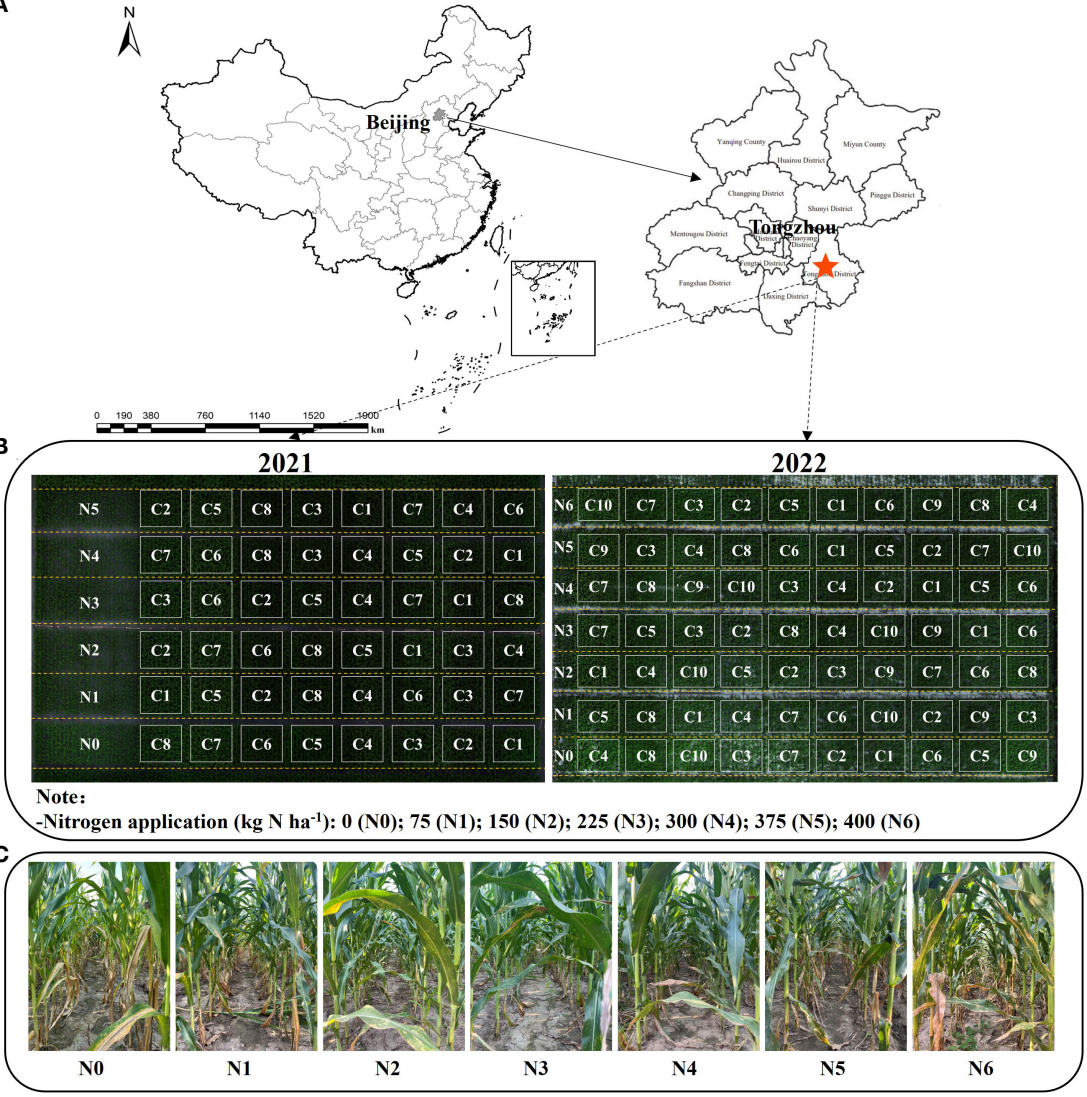
Geographical location of the study site **(A)**, Plot division of the experimental plots in 2021 and 2022 **(B)**. Canopy conditions under seven nitrogen fertilizers (N) treatments on August 10, 2022 **(C)**.

In 2021 and 2022, a two-year field experiment was conducted at the Research Base of Beijing Academy of Agriculture and Forestry Sciences, located in the International Seed Industry Science and Technology Park in Tongzhou District, Beijing, China (116°41′2″ E, 39°41′50″ N) (**Figure 1**). The study site is characterized by a typical warm temperate semi-humid continental monsoon climate, with an average annual temperature, precipitation, and sunshine hours of 13.8°C, 570.1mm, and 2396.2h, respectively. The two-year maize growing seasons spanned from June 1 to September 25, 2021, and from June 20 to September 30, 2022, respectively. Artificial thinning of seedlings is performed during the seedling stage. Local field management practices are followed, including the application of herbicides and insecticides as necessary, and sufficient rainfall occurs throughout the growing season.

**Table 1** presents the key details of the two tested cultivars, fertilization practices, and soil properties. The nitrogen fertilizer application was divided into two stages, with 50% applied as a base fertilizer before sowing and the remaining 50% applied at the jointing stage. To gather comprehensive information on canopy structure and LCC, Experiment 1 included 8 hybrid varieties and 6 nitrogen application treatments (0, 75, 150, 225, 300, and 375 kg N ha^-1^, denoted as N0—N5). Experiment 2 encompassed 10 hybrid varieties and 7 nitrogen application rates (0, 75, 150, 225, 300, 375, and 400 kg N ha^-1^, denoted as N0-N6). C1 to C10 correspond to 10 distinct maize varieties used in experiments 1 and 2, respectively. These varieties include Jingke999, Xianyu335, MC121, Dika159, Jingnongke728, Liangyu99, MC812, Zhengdan958, Jingnongke828, and Jingkeqingzhu516 (as shown in **Figure 1B**). Both experiments followed a split-plot design, with nitrogen fertilizer treatment as the main plot and varieties randomly distributed within the nitrogen fertilizer treatment, repeated 3 times. The planting method employed equal row spacing with 60 cm between rows, resulting in a planting density of 60,000 plants ha ^-1^. The total area of the experimental plots was approximately 0.48 ha. Data from the first year of Experiment 1 were utilized for model validation, while data from Experiment 2 were employed for model construction and calibration purposes.

**Table 1:** Basic information regarding two field experiments.

Experiment No.	Year	Treatments	Plots	Soil characteristics
Exp.1	2021	Cultivar: Jingke999, Xianyu335, MC121, Jingnongke728, Liangyu99, MC812, Zhengdan958, Jingnongke828. N application (kg N ha^-1^): 0, 75, 150, 225, 300, 375	48	Type: brown sandy Organic matter: 17.03 g kg^-1^ Total N: 1.08 g kg^-1^ Olsen-P: 0.067 g kg^-1^ Available-K: 0.241 g kg^-1^
Exp.2	2022	Cultivar: Jingke999, Xianyu335, MC121, Dika159, Jingnongke728, Liangyu99, MC812, Zhengdan958, Jingnongke828, Jingkeqingzhu516. N application (kg N ha^-1^): 0, 75, 150, 225, 300, 375, 400	70	Type: brown sandy Organic matter: 20.5 g kg^-1^ Total N: 1.35 g kg^-1^ Olsen-P: 0.078 g kg^-1^ Available-K: 0.201 g kg^-1^

### 2.2 Data collection

#### 2.2.1 Relative chlorophyll content (SPAD values)

During the four key growth stages of maize, namely the 6th leaf fully expanded stage (V6), the 9th leaf fully expanded stage (V9), the silking stage (R1), and the blister stage (R2), SPAD values for fully expanded leaves were obtained. From each plot, five plants with similar growth were selected, and their SPAD values were measured using a Minolta SPAD-502 chlorophyll meter manufactured in Japan. To determine the canopy SPAD value, record the SPAD values of all fully unfolded leaves along the main stem, starting from the bottom and moving towards the top. After completing all leaves measurements, calculate the average SPAD value of all leaves, which will serve as the canopy SPAD value. To ensure accuracy, each leaf was divided into intervals of 20% of its length, with measurements taken at 0-20%, 20-40%, 40-60%, 60-80%, and 80-100% of the leaf length. The SPAD values for each interval were determined accordingly. The results obtained from each plot were averaged to obtain representative measurements.

#### 2.2.2 UAV multispectral imagery acquisition and processing

**Figure 2:**
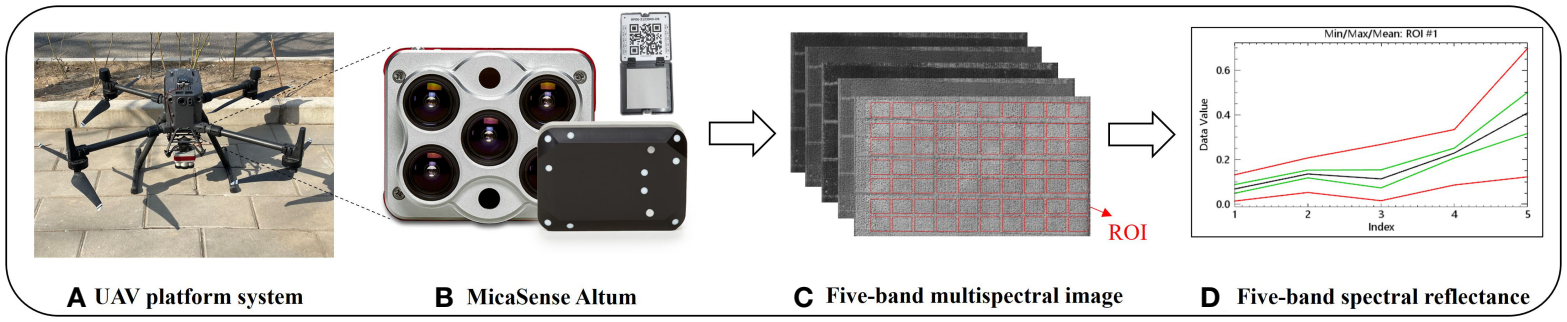
Unmanned aerial vehicle (UAV) multi-spectral system. **(A)** UAV platform, **(B)** MicaSense Altum camera and reflection calibration panel, **(C)** Single-band multi-spectral image and **(D)** Single-band reflectance extraction (The red line signifies the highest and lowest reflectance values among the five bands within the region of interest.; The green line represents the average reflectivity ± standard deviation of the five bands within the ROI and the black line represents the average reflectance of the five bands within the ROI).

During the 2022 growing season, UAV multispectral images were captured at four growth stages: V6, V9, R1, and R2, which coincided with the measurement of maize SPAD values. The UAV platform utilized a DJI M300 RTK multi-rotor UAV (SZ DJI Technology Co. LTD., Shenzhen, China), equipped with a Micasense Altum multispectral camera (AgEagle Sensor Systems Inc., Wichita, Kansas, USA) (**Figure 2**). The camera allows simultaneous data collection in five bands, including visible light (red, green, and blue), near-infrared, and red-edge bands. The center wavelength and bandwidth information for each band is provided in **Table 2**. The image resolution for each band is 2064×1544, with a field of view of 48°×36.8° and a focal length of 8mm. To ensure high-quality image stitching, the UAV flew at a height of 50m above the ground at a speed of 4m/s, the multispectral image has a spatial resolution of 1.5cm/pixel, overlap rates of 75% and 80% were set for the forward and side directions respectively. The entire research area can be covered in about 10 minutes per flight mission, resulting in the collection of approximately 1,240 multispectral images. A standard gray board with a constant reflectance is positioned along the flight path, ensuring it’s captured by the camera. It is utilized to calibrate the original multispectral image, generating a reflectance image. The multispectral data were captured under clear, cloudless conditions between 11:00 am and 2:00 pm. Using Pix4Dmapper (Pix4D S.A., Lausanne, Switzerland), multispectral image preprocessing was conducted to generate corrected spectral reflectance (with reflectance correction). Each output file was saved as a high-resolution TIFF image. Subsequently, ENVI 5.3 (HARRIS geospatial, Wokingham, UK) software was employed to merge the TIFF images from multiple bands into a 5-band reflectance TIFF image. A specific area of 100*100 pixels was selected as the region of interest (ROI) and save all plot ROIs as an XML file (**Figure 2C**). To ensure the consistency of ROIs across different growth stages, we register images from various time periods by relying on pre-arranged control points on the ground. Finally, the average reflectance of the pixels within each ROI was then extracted to represent the plots reflectance. The above steps follow the processing flow proposed by Mesas-Carrascosa and Li et al. (Mesas-Carrascosa et al., 2015; Li et al., 2023).

**Table 2:** MicaSense Altum multispectral camera parameters.

Waveband	Central wavelength/nm	Spectral bandwidth/nm
Blue	475	32
Green	560	27
Red	668	14
Red edge	717	12
Near infrared	842	48.8

#### 2.2.3 Vegetation indices extraction

The vegetation index is calculated based on UAV multispectral images. It is a linear or nonlinear combination of multiple spectral bands used to replace the band reflectance. Previous studies have established a direct correlation between LCC and leaf reflectance, which has subsequently spurred the development of multiple VIs (Narmilan et al., 2022). In this study, wide band VIs was calculated using multispectral images, and 17 well-established VIs related to chlorophyll were employed to establish the correlation between these indices and LCC during different growth stages (see **Table 3**).

**Table 3:** Summary of vegetation index selected in this study.

Vegetation Index	Name	Formula	Reference
Normalized difference vegetation index	NDVI	(Rnir − Rred)(Rnir + Rred)	(Li Z. et al., 2022)
Normalized Difference Chlorophyll Index	NDCI	(Rre−Rred)/(Rre+Rred)	(Mishra and Mishra, 2012)
Normalized difference red edge index	NDRE	(Rnir − Rre)/(Rnir+Rre)	(Li et al., 2015)
Green NDVI	GNDVI	(Rnir − Rgreen)/(Rnir + Rgreen)	(Gitelson et al., 2003)
Plant Pigment ratio	PPR	(Rgreen − Rblue)/(Rgreen+Rblue)	(Wang et al., 2004)
Canopy chlorophyll content	CCCI	(NDRE − (NDREmin)/(NDREmax−NDREmin)	(Fitzgerald et al., 2006)
MERIS Terrestrial Chlorophyll Index	MTCI	(Rnir − Rre)/(Rre−Rred)	(Dash and Curran, 2007)
Simple ratio	SR	Rnir/Rred	(Xue et al., 2004)
Red−edge chlorophyll index	CIred−edge	(Rnir/Rre) − 1	(Gitelson, 2005)
Green chlorophyll index	CIgreen	(Rnir/Rgreen) − 1	(Gitelson et al., 2003)
Transformed Chl absorption in reflectance index	TCARI	3*[(Rre − Rred) − 0.2*(Rre − Rgreen)(Rre/Rred)]	(Haboudane et al., 2002)
Triangular vegetation index	TVI	60*(Rnir − Rgreen) − 100*(Rred−Rgreen)	(Yang et al., 2022)
mTVI (red-edge)	mTVI	60*(Rnir − Rgreen) − 100*(Rre−Rgreen)	(Broge and Leblanc, 2001)
Modified chlorophyll absorption ratio index	MCARI	(Rre − Rred) − 0.2*(Rre−Rgreen) * (Rre/Rred)	(Daughtry, 2000)
mNDblue	mNDblue	(Rblue − Rre)/(Rblue + Rnir)	(Jay et al., 2017)
Enhanced vegetation index	EVI	2.5*(Rnir − Rred)/(Rnir + 6*Rred − 7.5*Rblue+1)	(Peng et al., 2017)
Difference Vegetation Index	DVI	Rnir − Rred	(Li Z. et al., 2022)

### 2.3 Construction of Lorentz peak distribution function based on leaf SPAD values

In this study, the vertical variation of maize leaf SPAD values was simulated using the Lorentz peak distribution function. The original definition of the Lorentz peak distribution function is as follows:


(1) $$y=\frac{y_{m}}{1+{\left(\frac{x-x_{0}}{b}\right)}^{2}}$$

this function has three parameters: *y_m_*, the peak of the curve; *b*, the slope of the curve; *x_0_*, the independent variable corresponding to the peak of the curve. Considering that the main purpose of this study is to fit the vertical non-uniform curve of the SPAD values of maize canopy leaves, formula (1) is redefined as:


(2) $$SPAD=\frac{SPAD_{m}}{1+{\left(\frac{n-n_{m}}{b}\right)}^{2}}$$

where *SPAD_m_* represents the maximum SPAD values, *n* represents different leaf positions, *n_m_* represents the leaf position corresponding to the maximum SPAD values, *b* represents the slope of the curve.

### 2.4 Data analysis

The leaf SPAD value data collected over the two-year experiment constituted the dataset for constructing the Lorentz peak distribution function. Select the leaves dataset from Experiment 2 as the calibration set for the Lorentz peak function (V6, n=420; V9, n=630; R1, n=1010; R2, n=950), and the leaves dataset from Experiment 1 as the validation set for the model (V6, n=36; V9, n=54; R1, n=82; and R2, n=78). In this verification process, SPAD values of leaves from different varieties treated with each nitrogen fertilizer in Experiment 1 were subjected to averaging.

To assess the UAV platform’s sensitivity to SPAD values at different leaf positions in the maize canopy, the Pearson correlation coefficient (r) Equation 6 was utilized to evaluate the correlation between VIs and SPAD values at various leaf positions. The p-value was then employed to determine statistical significance, with a threshold of p< 0.05 considered significant. Lastly, the VIs extracted through UAV multispectral analysis were employed to formulate a linear regression model incorporating sensitive leaf position SPAD values and overall canopy SPAD values (average SPAD values of all leaves). Evaluation metrics, including the coefficient of determination (R^2^) Equation 3, root mean square error (RMSE) Equation 4, and normalized root mean square error (nRMSE) Equation 5, were selected to quantify the explained variation, and assess model performance. The calculation formula is as follows:


(3) $$R^{2}=1-\frac{\sum_{i=1}^{n}{\left(y_{i}-P_{i}\right)}^{2}}{\sum_{i=1}^{n}{\left(y_{i}-\bar{y}\right)}^{2}}$$

(4) $$RMSE=\sqrt{\frac{\sum_{i=1}^{n}{\left(y_{i}-P_{i}\right)}^{2}}{n}}$$

(5) $$nRMSE=\frac{RMSE}{\left(y_{max}-y_{min}\right)}$$

where $P_{i}$ represents the predicted value, $\bar{y}$ represents the average value of the measured value, $y_{i}$ represents the measured value, and *n* represents the number of samples, and $y_{max}$ and $y_{min}$ are the maximum and minimum sample values.


(6) $$r=\frac{\sum_{i=1}^{n}(X_{i}-\bar{X})(Y_{i}-\bar{Y})}{\sqrt{\sum_{i=1}^{n}{\left(X_{i}-\bar{X}\right)}^{2}}\sqrt{\sum_{i=1}^{n}{\left(Y_{i}-\bar{Y}\right)}^{2}}}$$

where r is the correlation coefficient between variable $X\;=\left\{X_{i}\right\}\left(1\leq \text{i}\leq \text{n}\right)$ and variable $Y=\;\left\{Y_{i}\right\}\left(1\leq \text{i}\leq \text{n}\right)$ , r > 0 indicates that variable X is positively correlated with Y , and r < 0 indicates that both r = 0 indicates that the two are not correlated; $X_{i}$ is the measured value of the variable X  at the i-th position; $\bar{X}$ is the mean value of the variable X ; $Y_{i}$ is the measured value of the variable Y at the i-th position; $\bar{Y}$ is the mean value of variable Y ; n is the number of variable X or Y (this study refers to the number of leaves and the number of samples in the experimental plot).

## 3 Result

### 3.1 Vertical distribution of SPAD values within the canopy

**Figure 3:**
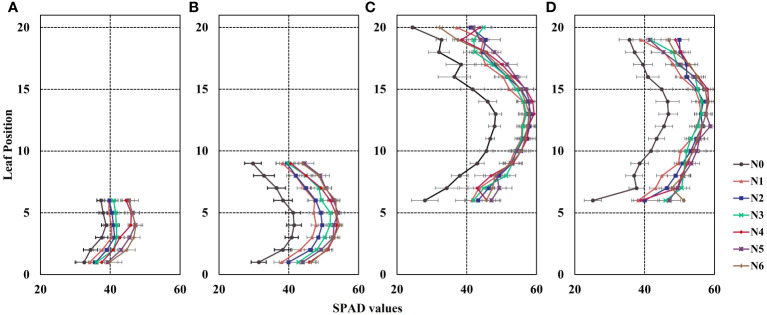
Vertical distribution of leaf SPAD values in maize canopy during the 2022 growing season. The Y-axis represents leaf position. **(A)** the 6th leaf fully expanded stage (V6), **(B)** the 9th leaf fully expanded stage (V9), **(C)** the silking stage (R1), and **(D)** the blister stage (R2). Error bars show standard deviation. In the R1 and R2 growth stages, the lower leaves exhibit senescence and yellowing, while most varieties experience leaf shedding at the top in the R2 stage. To ensure data consistency, the leaf counts for R1 and R2 are 6-20 and 6-19, respectively.

Based on the data presented in **Figure 3**, it is evident that the distribution of canopy SPAD values in maize varied across different growth stages. The distribution pattern exhibited a non–uniform trend, characterized by an initial increase followed by a decrease from the bottom to the top of the canopy, resembling a bell-shaped curve. Specifically, during the V6 growth stage, the highest SPAD values under different nitrogen fertilizer treatments (N1–N6) were primarily observed in the 2nd and 3rd leaf positions (**Figure 3A**). In the V9 stage, the maximum SPAD values were found in the 4th, 5th, and 6th leaf positions (**Figure 3B**). As for the R1 and R2 stages, the peak SPAD values were distributed in the ear position leaf and the two leaves above and below the ear position (**Figures 3C, D**).

**Figure 4:**
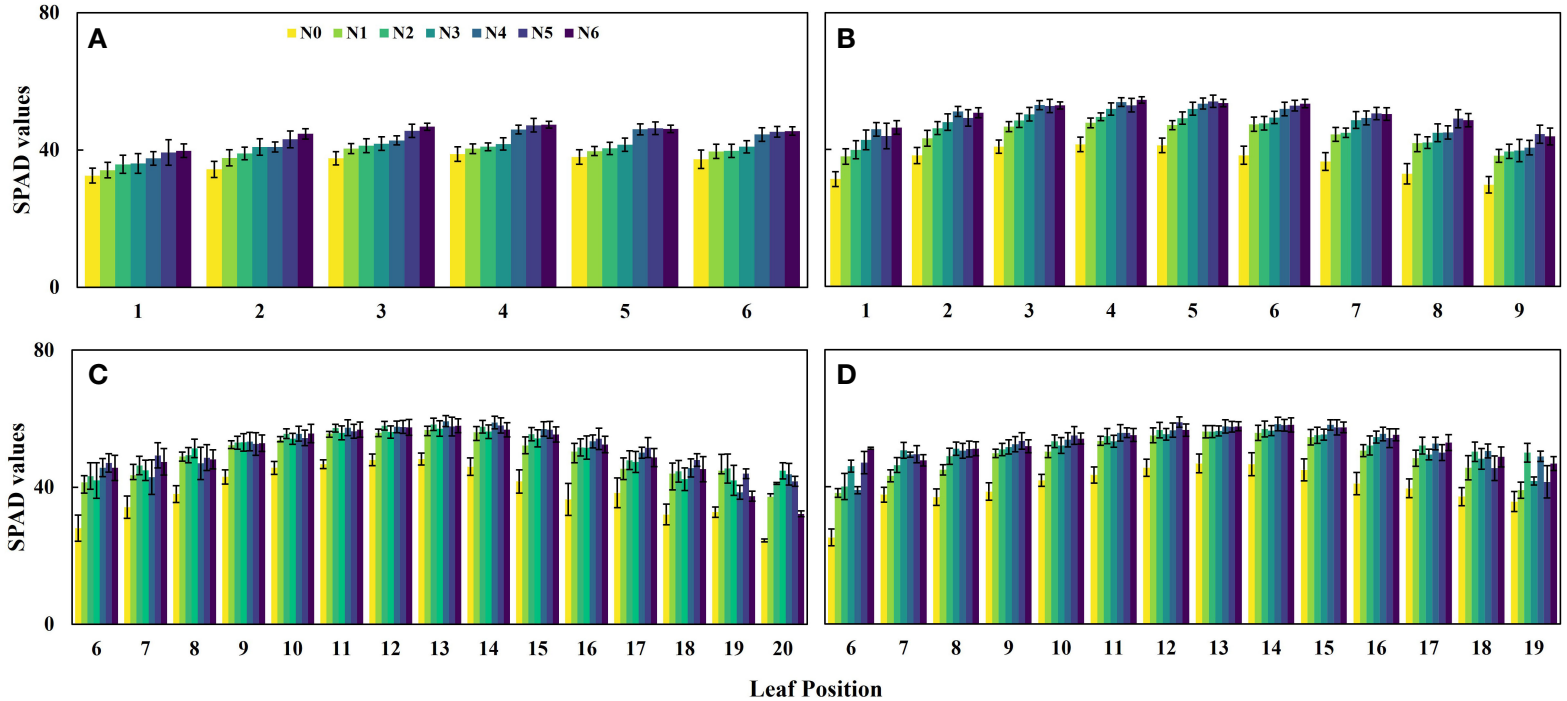
Changes of SPAD value in the same leaf position under different nitrogen application rates. **(A)** V6 stage, **(B)** V9 stage, **(C)** R1 stage, and **(D)** R2 stage.

Additionally, the results depicted in **Figure 3** indicate that the SPAD values of maize canopies treated without nitrogen application (N0) were significantly lower than those subjected to nitrogen treatment across all four growth stages. Notably, the discrepancy in canopy SPAD values between different nitrogen application rates was more prominent during the vegetative growth stages (V6 and V9) and diminished during the reproductive growth stages (R1 and R2). This observation can be attributed to the nutrient transfer mechanism in maize plants, where nutrients are transported from the lower parts to the upper parts during the later growth stages to support photosynthesis in the upper leaves. Consequently, the lower leaves of the maize exhibit progressive senescence and yellowing, as evident in the experimental plots (**Figures 3C, D**). Furthermore, during the V6 and V9 stages, the SPAD values of the same leaf position displayed an upward trend with increasing nitrogen application (**Figures 4A, B**). However, during the R1 and R2 stages, the SPAD values of the same leaf position exhibited an initial increase followed by a decrease with increasing nitrogen application.

### 3.2 Modeling and validation of canopy SPAD values based on the Lorentz peak distribution function

**Figure 5:**
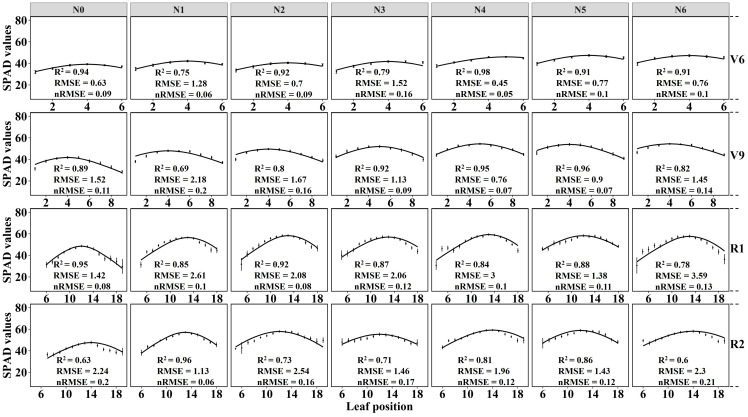
Lorentz peak curve fitting under different nitrogen application rates at four growth stages. The solid line represents the fitted curve, the scatter represents the true SPAD value, and the error line represents the standard deviation.

The results displayed in **Figure 5** demonstrate the fitting of crown SPAD values for maize at four growth stages using the Lorentz peak distribution function Equation 2. The findings indicate that the Lorentz peak distribution function effectively fits the distribution of crown SPAD values at these stages, with R^2^ and RMSE values as follows: V6 (R^2^: 0.75 – 0.98, RMSE: 0.45 – 1.28); V9 (R^2^: 0.69 – 0.96, RMSE: 0.9 – 2.18); R1 (R^2^: 0.78 – 0.95, RMSE: 1.42 – 3.59); and R2 (R^2^: 0.6 – 0.96, RMSE: 1.13 – 2.3). Notably, the highest fitting accuracy was observed for the no nitrogen treatment (N0) in the V6 stage (R^2^ = 0.94, RMSE = 0.63); the N5 treatment in the V9 stage showed the highest fitting accuracy (R^2^ = 0.96, RMSE = 0.9); the N0 treatment in the R1 stage exhibited the highest fitting accuracy (R^2^ = 0.95, RMSE = 1.42); and the N1 treatment in the R2 stage displayed the highest fitting accuracy (R^2^ = 0.96, RMSE = 1.13). Except for the V9 stage, the Lorentz peak distribution function effectively fits the leaf SPAD values under both no nitrogen and low nitrogen conditions.

**Figure 6:**
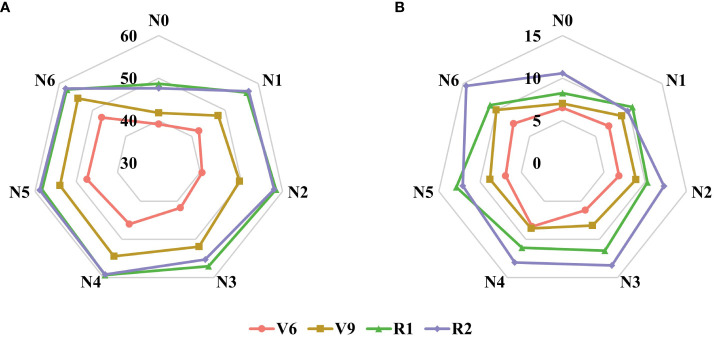
Changes of parameters *SPAD_max_* **(A)** and *b* **(B)** at different growth stages and fertilization conditions in the growing season of maize in 2022.

**Figure 7:**
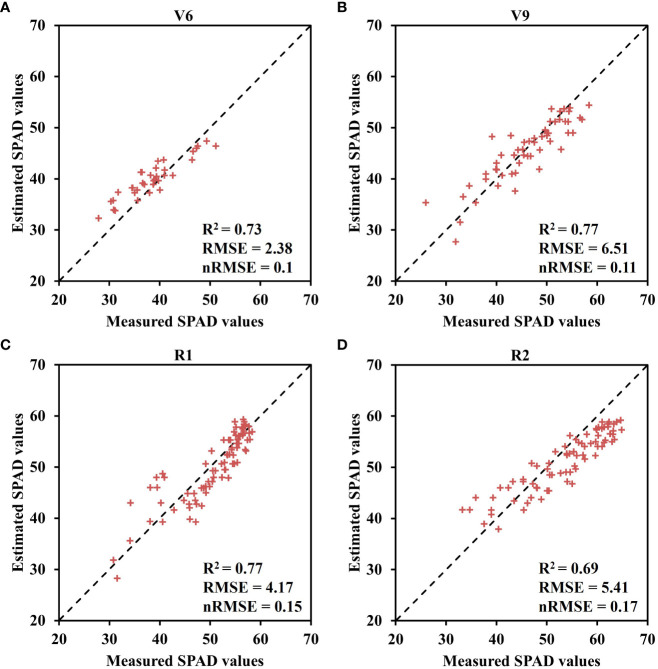
Verification results of fitting SPAD value to Lorentz peak distribution function at four growth stages in 2021 growing season. **(A)** V6 stage, **(B)** V9 stage, **(C)** R1 stage, and **(D)** R2 stage.

Moreover, **Table 4** presents the statistical results of the three function parameters: *SPAD_m_*, *n_m_*, and *b* values, under different nitrogen application treatments and growth stages. The maximum SPAD values (*SPAD_m_*) and leaf positions (*n_m_*) during the V6 and V9 stages are primarily concentrated in the 4th and 5th leaves, while during the R1 and R2 stages, the *n_m_* are mainly distributed in the 12th and 13th leaves. *SPAD_m_* and *b* values exhibit similar patterns of variation across different nitrogen application treatments and growth stages (**Figure 6**). The results indicate a positive correlation between both *SPAD_m_* and *b* parameters with nitrogen application rate and growth stage, implying that increasing nitrogen fertilizer application can enhance *SPAD_m_* and *b* values. Notably, there is a significant difference between nitrogen application and no nitrogen application treatments. As the growth stage progresses, *SPAD_m_* and *b* gradually increase. While there is no significant change in *SPAD_m_* from the R1 to R2 stage, the difference in *b* among the four growth stages is significant. Based on the fitting results obtained from the model, the model was further validated using experimental data collected in 2021 (**Figure 7**). The validation results of the Lorentz peak distribution function, based on the measured data sets from the four growth stages (V6, V9, R1, and R2), demonstrate good estimation accuracy. The highest accuracy was observed in the V9 and R1 stages (V9: R^2^ = 0.77, RMSE = 6.51; R1: R^2^ = 0.77, RMSE = 4.17), followed by the V6 stage (R^2^ = 0.73, RMSE = 2.38). Slightly lower accuracy was observed in the R2 stage (R^2^ = 0.69, RMSE = 5.41).

**Table 4:** The statistical results of Lorentz peak distribution function parameters.

N treatment	V6			V9			R1			R2		
	*SPAD_m_*	*n_m_*	*b*	*SPAD_m_*	*n_m_*	*b*	*SPAD_m_*	*n_m_*	*b*	*SPAD_m_*	*n_m_*	*b*
N0	39.16	4	6.51	41.80	4	7.01	48.69	12	8.24	47.62	14	10.58
N1	42.12	4	6.99	47.94	4	8.92	56.58	13	10.56	57.12	13	9.83
N2	40.48	4	6.83	49.55	4	8.87	58.37	13	10.26	57.92	12	12.27
N3	41.75	4	6.18	51.92	5	8.17	57.06	13	11.49	55.28	12	13.40
N4	46.00	5	8.32	54.40	5	8.55	59.33	13	11.09	59.20	14	13.02
N5	47.40	4	6.90	53.89	4	8.77	58.29	12	12.87	58.86	12	12.08
N6	47.22	4	7.42	54.40	4	10.03	57.77	13	10.94	58.11	14	14.56

### 3.3 Identification and selection of VIs with high sensitivity for each growth stage

A linear regression model was constructed using the 2021 field experiment data and UAV multispectral data to establish the relationship between canopy SPAD values and VIs. The fitting accuracy of the canopy SPAD values and VIs was assessed using R2 and RMSE. The results demonstrate consistent strong correlations between canopy SPAD values and VIs across different growth stages. The top 5 VIs with strong correlations in each stage, including CCCI, MTCI, CIred-edge, NDRE, and GNDVI, are presented in **Table 5**. Moreover, the correlation coefficients between VIs and crown SPAD values varied across stages: V6 (r: 0.583 – 0.665), V9 (r: 0.735 – 0.814), R1 (r: 0.777 – 0.788), and R2 (r: 0.802 – 0.866). The results indicate an increasing correlation between VIs and crown SPAD values as the growth stage progresses. Specifically, CCCI exhibited the highest correlation coefficients in V9 and R2, with r values of 0.814 (RMSE = 3.17) and 0.866 (RMSE = 2.59), respectively. CCCI also performed well in V6 and R1, with r values of 0.662 (RMSE = 3.57) and 0.783 (RMSE = 3.16), respectively. Therefore, considering convenience for further research, CCCI was selected as the optimal VI for subsequent investigation.

**Table 5:** Analyzing the correlation between the vegetation index and SPAD value across four phenological stages.

Phenological Period	N0.	VIs	Correlation coefficient	RMSE
V6	1 2 3 4 5	MTCI CCCI mTVI CIred-edge NDRE	0.665 0.662 0.590 0.584 0.583	3.57 3.57 3.85 3.87 3.88
V9	1 2 3 4 5	CCCI MTCI NDRE GNDVI CIgreen	0.814 0.783 0.744 0.743 0.735	3.17 3.39 3.64 4.41 4.47
R1	1 2 3 4 5	CIred-edge MTCI CCCI CIgreen NDRE	0.788 0.787 0.783 0.780 0.777	3.12 3.13 3.16 3.17 3.19
R2	1 2 3 4 5	CCCI MTCI NDRE CIred-edge GNDVI	0.866 0.855 0.812 0.808 0.802	2.59 2.68 3.02 3.05 3.09

“N0.” indicates the ranking order of the correlation coefficient.

### 3.4 Selection of sensitive leaf positions and establishment of an inversion model for predicting SPAD values

**Figure 8:**
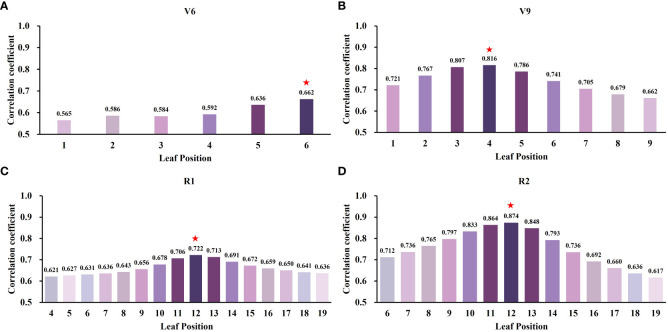
Sensitivity of leaf position SPAD to canopy spectral index at different growth stages. ★represents the most sensitive leaf position in each period. **(A)** V6 stage, **(B)** V9 stage, **(C)** R1 stage, and **(D)** R2 stage.

Based on the findings from the previous section, the CCCI was chosen as the optimal vegetation index for selecting sensitive leaf positions within the crown across the four growth stages, as depicted in **Figure 8**. The analysis revealed that in the V6 stage, the sixth leaf position displayed the highest correlation coefficient with CCCI (r = 0.662), while the first leaf position exhibited the lowest correlation coefficient (r = 0.565). The correlation coefficients gradually increased from the lower to the upper regions of the canopy. In the V9 stage, the fourth leaf position demonstrated the highest correlation coefficient (r = 0.816), whereas the ninth leaf position had the lowest correlation coefficient (r = 0.662). The correlation coefficients exhibited an ascending trend followed by a descending trend from the first to the ninth leaf position. For both the R1 and R2 stages, the twelfth leaf position exhibited the maximum correlation coefficients of 0.722 and 0.874, respectively. Furthermore, the correlation coefficients between different leaf positions and CCCI displayed a similar decreasing trend from the middle to the upper and lower regions of the canopy for both stages.

**Figure 9:**
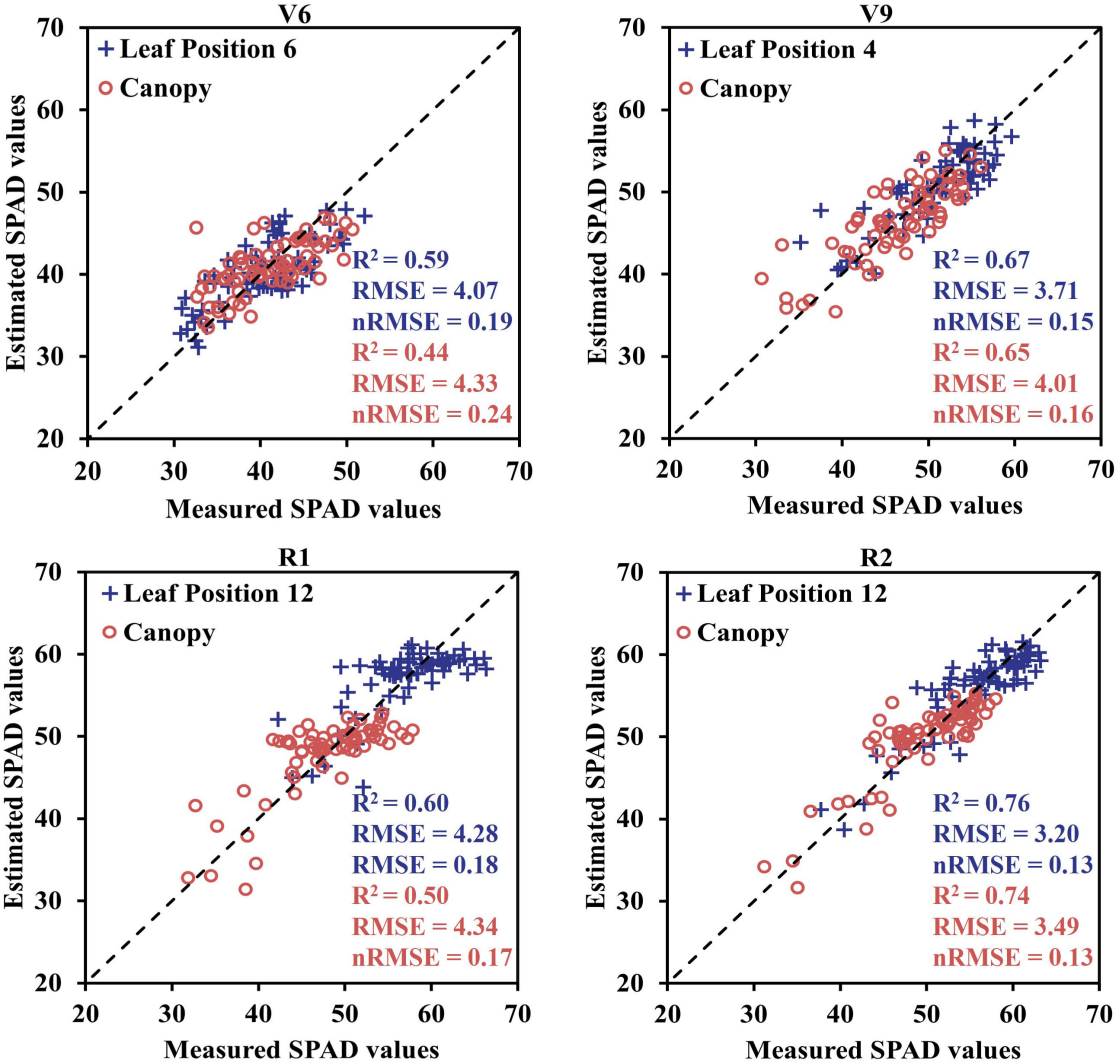
The estimation results of sensitive leaf position and canopy SPAD values at different growth stages were obtained by using unitary linear regression model.

**Figure 10:**
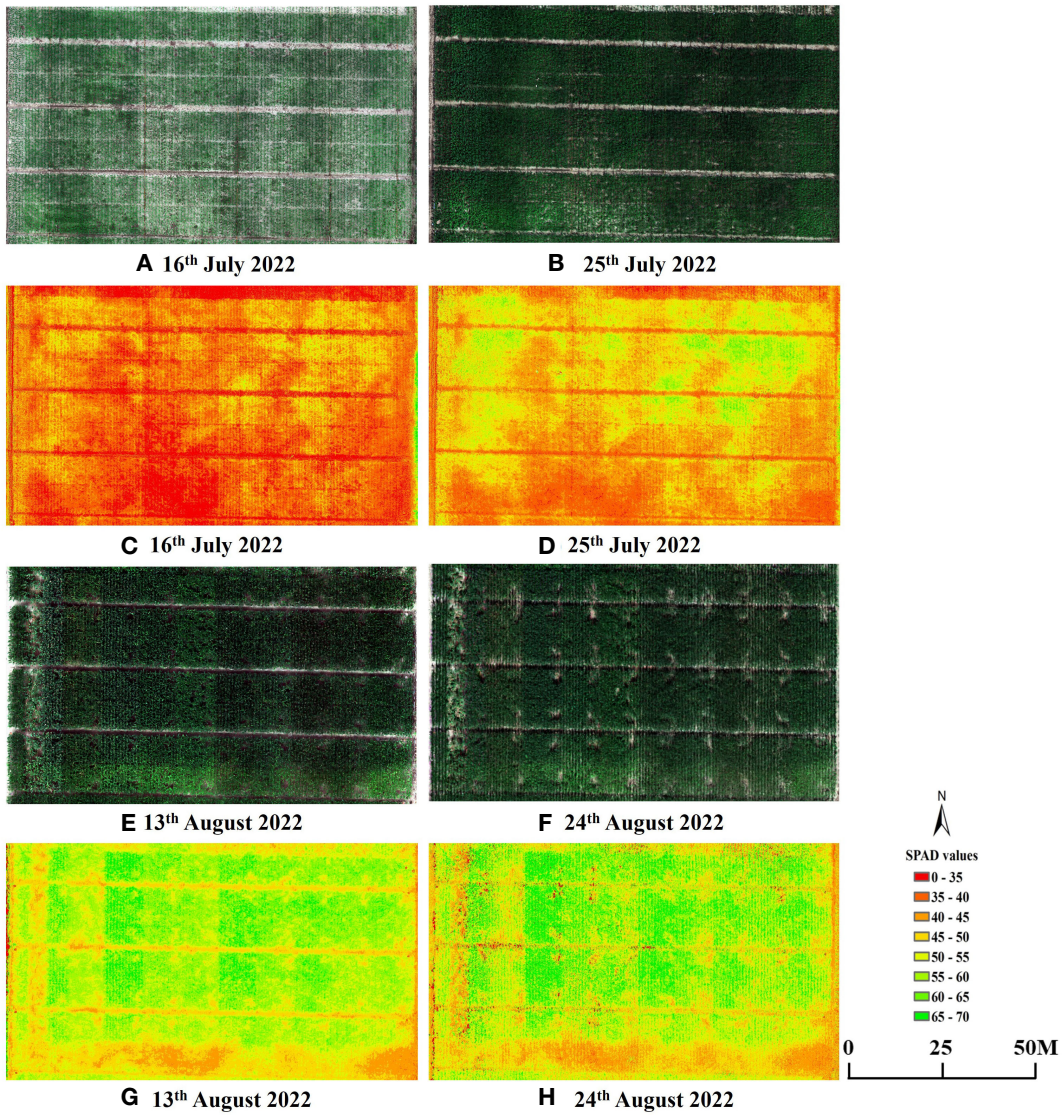
UAV RGB images and SPAD values maps for each plot. Examples presented are: **(A, C)** V6; **(B, D)** V9; **(E, G)** R1 and **(F, H)** R2.

Furthermore, to validate the effectiveness of estimating maize SPAD values using sensitive leaf positions, simple linear regression models were developed using these positions (V6: 6; V9: 4; R1: 12; R2: 12) and canopy SPAD values with VIs for the four growth stages. These models were then tested on two datasets. The relationship between the predicted and measured SPAD values, based on the CCCI model constructed using the two datasets and UAV-derived data, is presented in **Figure 9**. The estimation accuracy of SPAD values at the sensitive leaf positions in the four growth stages, based on UAV spectral data, was superior to that at the canopy scale. The R^2^ and RMSE values for the sensitive leaf positions at the four growth stages were 0.59, 0.67, 0.60, and 0.76, respectively, with corresponding RMSE values of 4.07, 3.71, 4.28, and 3.20. In contrast, the R^2^ and RMSE values for the canopy were 0.44, 0.65, 0.50, and 0.74, respectively, with corresponding RMSE values of 4.33, 4.01, 4.34, and 3.49. The estimation accuracy of the sensitive leaf positions in V6 and R1 was significantly better than that at the canopy scale, while the improvement effect was not as evident in the V9 and R2 stages. Additionally, **Figure 10** displays the spatiotemporal distribution of predicted SPAD values using UAV multispectral images, highlighting the variability of SPAD values in response to different treatments and growth stages.

## 4 Discussion

Crop chlorophyll remote sensing plays a crucial role in quantitative remote sensing of crops. Timely and accurate monitoring of crop canopy chlorophyll content is of great significance for agricultural management (Cabangon et al., 2011). Although the heterogeneity of vertical chlorophyll distribution in crop canopies has been confirmed by numerous studies (Shiratsuchi et al., 2006; Wu et al., 2021), there is still considerable room for improvement in the accuracy and mechanisms of remote sensing monitoring methods.

### 4.1 Characteristics of temporal and spatial heterogeneity in canopy SPAD values distribution of maize

This study first conducted experiments involving multiple growth stages and different nitrogen fertilization treatments to demonstrate the asymmetric curve distribution of SPAD values in maize canopy leaves along the vertical direction. The SPAD values were higher in the middle leaf position compared to the top and bottom layers (**Figure 3**), and this distribution pattern was consistent in both vegetative and reproductive growth stages, consistent with previous research findings (Ciganda et al., 2008; Winterhalter et al., 2012; Yang et al., 2022). Moreover, different nitrogen application treatments resulted in distinct canopy structure types (**Figure 3**), and the SPAD values of the same leaf position increased with higher nitrogen application rates (**Figure 4**). Nitrogen is an essential component of chlorophyll and highly mobile in plants, and increasing nitrogen fertilizer can enhance the nitrogen supply capacity of the soil to crops (Bonelli and Andrade, 2020; Zhang et al., 2021). Therefore, nitrogen fertilization significantly influences leaf SPAD values and alters the canopy’s distribution structure. Consequently, quantitatively simulating canopy leaf SPAD values should consider the fertilization level and different growth stages.

### 4.2 Performance of the Lorenz peak distribution function model

Based on the obtained results, the application of the Lorentz peak distribution function to fit the spatial distribution of maize canopy SPAD values demonstrated excellent performance across different growth stages and nitrogen application treatments (**Figure 5**). Among the four growth stages, the treatments that yielded the best model fitting were N0, N5, N0, and N1, respectively, with the Lorentz peak distribution function performing exceptionally well during the V6 stage. One key advantage of using this function is its ability to better represent the crucial structural parameters of the maize canopy through its three parameters (*SPAD_m_*, *n_m_*, and *b*). The research findings confirm that the distribution structure of maize canopy SPAD values follows an asymmetric curve, encompassing the maximum SPAD values, the leaf position with the highest SPAD values, and the gradient change of SPAD values at different leaf positions, represented by *SPAD_m_*, *n_m_*, and *b*. The nm values at different reproductive stages are 5, 5, 13, and 14, respectively (**Table 4**). During the early growth stage, nm is primarily concentrated in the upper-middle layer of the canopy because the upper leaves of maize are adept at capturing more light energy for photosynthesis, which fulfills the crop’s growth requirements (Ciganda et al., 2009). In the reproductive growth stages, nm predominantly appears in the three-ear leaves. As the functional leaves of maize, the nutrient index of the three-ear leaves directly influences grain growth and development. Therefore, the three-ear leaves intercept more light for photosynthesis to ensure the contribution of grain carbohydrates (Li et al., 2019). Additionally, both the *SPAD_m_* and *b* parameters exhibited an upward trend with increasing nitrogen application rate and growth stage (**Figure 6**), indicating that an augmented nitrogen application can modify the distribution structure of SPAD values in the maize canopy. Furthermore, as the growth stage advances, the disparity in SPAD values between different leaf layers becomes more pronounced, resulting in a steeper curve slope. Furthermore, this study employed field experiment data from 2021 to verify the constructed Lorentz peak distribution function across multiple years (**Figure 7**). All four growth stages yielded satisfactory verification results: V6, R^2^ = 0.73, RMSE = 2.38; V9, R^2^ = 0.77, RMSE = 6.51; R1, R^2^ = 0.77, RMSE = 4.17; R2, R^2^ = 0.69, RMSE = 5.41. These findings indicate the model’s robustness and applicability in different growing seasons. However, due to space limitations, this study did not establish a quantitative relationship between the model parameters and the two indicators of fertilization rate and phenological period. Future research can explore this aspect further.

### 4.3 Construction and application of UAV platform estimation model

VIs has been widely recognized as effective tools for rapid and nondestructive estimation of various crop canopy parameters (Palka et al., 2021). Therefore, in this study, 17 VIs that have been previously linked to canopy chlorophyll (**Table 3**) were selected, and the sensitivity of these VIs to canopy SPAD values was assessed using Pearson correlation coefficient analysis. The findings revealed that VIs such as CCCI, MTCI, and NDRE exhibited strong correlations with maize canopy.

SPAD values across the four growth stages (**Table 5**). These results align with previous research by C. S. T. Daughtry et al. (Daughtry, 2000) and Bin Wu et al. (Wu et al., 2021), emphasizing the significance of the red-edge band in establishing VI and chlorophyll models. Among the evaluated VIs, CCCI consistently demonstrated the best performance across different growth stages, which corroborates the findings of Davide Cammarano et al. (2011) and Fei Li et al. (2014). Notably, the leaf positions exhibiting the highest correlation between leaf SPAD values and CCCI varied across the four growth stages (**Figure 8**), highlighting the need to construct separate canopy VIs models for accurate estimation of leaf SPAD values based on growth stage. To further validate the advantages of using UAV spectral data for estimating SPAD values in sensitive leaf positions, field data and UAV canopy spectral data collected in 2022 were employed. The results revealed that the estimation accuracy of SPAD values at the four growth stages based on UAV multispectral data surpassed that at the canopy scale (**Figure 9**). Specifically, the R^2^ values increased by 34% for V6, 3% for V9, 20% for R1, and 3% for R2, respectively. These findings emphasize that the vertical distribution characteristics of maize canopy structure significantly impact the UAV-based SPAD estimation model, indicating that the estimation of crop canopy indicators using UAV cannot assume a uniform distribution throughout the canopy.

In this study, the utilization of the Lorentz peak distribution function facilitated the quantitative simulation of SPAD values for different leaf positions within the maize canopy. By integrating the spatiotemporal distribution characteristics of canopy SPAD values with the UAV multispectral estimation model, the accuracy of the UAV remote sensing model for canopy SPAD values was significantly improved. However, there are still some issues that need to be improved in this study. 1. Multi source data fusion, previous studies have shown that fusing multiple sources of data (spectral data, texture information, morphological parameters) can effectively improve model accuracy. In the future, low-cost high-definition digital cameras can be used to obtain multi-source data to verify the feasibility of this research method (Liu et al., 2022a; Liu et al., 2022b); 2. Cross scale applications, UAV remote sensing has the advantages of strong mobility and high resolution, but there are still certain limitations in its application in large-scale environments due to the large environment (weather, wind speed). So future research should focus on how to combine UAV remote sensing with satellite remote sensing (Lou et al., 2021); 3. Model algorithms, traditional machine learning algorithms such as partial least squares and random forests, as well as commonly used deep learning and transfer learning, have been widely used in remote sensing models, but they require a large amount of data. In the future, data from different experimental points can continue to be collected, and machine learning algorithms can be combined to improve the accuracy of the model (Fu et al., 2023).

## 5 Conclusion

This work incorporates the spatiotemporal distribution characteristics of maize leaf SPAD values into an UAV remote sensing estimation model. By constructing a vertical distribution function for maize canopy SPAD values, we have effectively enhanced the accuracy of the UAV remote sensing model for canopy SPAD values. The results show that:

1. The canopy SPAD values of maize during critical growth stages exhibit a non-uniform vertical distribution pattern resembling a “bell-shaped” curve. The canopy SPAD values of the non-fertilized treatment are significantly lower than those of the fertilized treatment.
2. The fitting of maize canopy SPAD values was achieved based on the Lorenz peak distribution function, and the obtained results were validated using inter-annual data (**Figure 7**).
3. The accuracy of estimating the UAV multispectral model, constructed based on sensitive leaf position SPAD values, surpasses that of the canopy-scale model, with respective improvements in R^2^ values for V6 (34%), V9 (3%), R1 (20%), and R2 (3%).

The findings of this study highlight that employing quantitative modeling of canopy indicators can substantially enhance the precision of remote sensing estimation. However, it is important to note that the validation of this approach was conducted at a single ecological site and with a single crop. Further research is necessary to establish the applicability of this method across multiple ecological sites and other crops. Additionally, there is an opportunity for further exploration of coupling canopy model parameters with models such as radiative transfer, which can contribute to more effective guidance of agricultural production management.

## Data availability statement

The raw data supporting the conclusions of this article will be made available by the authors, without undue reservation.

## Author contributions

BC: Conceptualization, Data curation, Investigation, Methodology, Validation, Writing – original draft, Writing – review & editing. GH: Supervision, Writing – review & editing. XL: Funding acquisition, Supervision, Writing – review & editing. SG: Methodology, Supervision, Validation, Investigation, Software, Writing – review & editing. WW: Funding acquisition, Project administration, Investigation, Software, Writing – review & editing. GW: Supervision, Validation, Investigation, Writing – review & editing. WC: Supervision, Validation, Investigation, Writing – review & editing. XG: Conceptualization, Formal Analysis, Funding acquisition, Project administration, Resources, Writing – review & editing. CZ: Conceptualization, Formal Analysis, Funding acquisition, Project administration, Resources, Supervision, Writing – review & editing.
